# Evidence of limited carbon sequestration in soils under no-tillage systems in the Cerrado of Brazil

**DOI:** 10.1038/srep21450

**Published:** 2016-02-24

**Authors:** Marc Corbeels, Robelio Leandro Marchão, Marcos Siqueira Neto, Eliann Garcia Ferreira, Beata Emöke Madari, Eric Scopel, Osmar Rodrigues Brito

**Affiliations:** 1 Agro-ecology and Sustainable Intensification of Annual Crops, CIRAD, Avenue Agropolis, 34398 Montpellier cedex 5, France; 2 Embrapa-Cerrados, PO Box 8233, 73301-970 Planaltina, DF, Brazil; 3 Centro de Energia Nuclear na Agricultura, Universidade de São Paulo, PO Box 96, 13400-970, Piracicaba, SP, Brazil; 4 Universidade Estadual de Londrina, Londrina, Brazil; 5 Embrapa Arroz e Feijão PO Box 179, 75375-000, Santo Antônio de Goiás, GO, Brazil

## Abstract

The Brazilian government aims at augmenting the area cropped under no-tillage (NT) from 32 to 40 million ha by 2020 as a means to mitigate CO_2_ emissions. We estimated soil carbon (C) sequestration under continuous NT systems in two municipalities in the Goiás state that are representative of the Cerrado. A chronosequence of NT fields of different age since conversion from conventional tillage (CT) was sampled in 2003 and 2011. Soil C levels of native Cerrado and pasture were measured for comparison. After about 11 to 14 years, soil C stocks under NT were highest and at the levels of those under natural Cerrado. Average annual rates of soil C sequestration estimated using the chronosequence approach were respectively 1.61 and 1.48 Mg C ha^−1^ yr^−1^ for the 2003 and 2011 sampling, and were higher than those observed using repeated sampling after eight years. The diachronic sampling revealed that the younger NT fields tended to show higher increases in soil C stocks than the older fields. Converting an extra 8 million ha of cropland from CT to NT represents an estimated soil C storage of about 8 Tg C yr^−1^ during 10 to 15 years.

At the Copenhagen Climate Change Conference of 2009 the Brazilian government committed to mitigation actions leading to projected reductions in its greenhouse gas emissions of 36 to 39% by 2020^1^. Brazil is one of the major greenhouse gas emitters in the world with historically more than half of its emissions originating from deforestation for agricultural land. From 2005 to 2010 reduced rates of deforestation resulted in circa 40% decrease in the total Brazilian emissions^2^. Today, emissions from agriculture, originating from cattle ranching and to a lesser extent from chemical fertilizer use, are at least equivalent to those from deforestation. Beside the continued efforts to control and reduce emissions from deforestation, one of the mitigation measures is the implementation of strategies that maintain and increase stocks of organic carbon (C) in agricultural soils. The adoption of no-tillage (NT) systems in croplands is considered as an effective way to achieve this. Within its ‘Action Plan for Low Carbon Agriculture’ launched in 2010, the Brazilian government aims at augmenting the area cropped under NT from 32 to 40 million ha by 2020^3^. Several studies suggest that a change from conventional tillage (CT) to NT cropping systems leads to an increase in soil C, especially if this occurs with intensification of crop production^4^,^5^,^6^,^7^.

In Brazil, the Cerrado region is regarded as the target region for the efforts in mitigating CO_2_ emissions through the adoption of NT systems^8^. The Cerrado occupies about 23% of the Brazilian territory or about two million km^2^ in the central part of the country^9^. The region has experienced a rapid expansion of large-scale commercial agriculture since the early 1970s, when governmental policies were put in place designed to increase production of commodities for export as a response to the increased global demand for soybean and meat^10^,^11^. From a total of 136 million ha suitable for agriculture, about 40 to 50% had been converted to pasture or cropland by 2002^12^,^13^. The region accounts for 55% of Brazil’s total grain production (www.conab.gov.br). Oxisols (USDA soil taxonomy) are the dominant soils covering about 45% of the region^14^.

Farmers in the Cerrado region started to adopt NT systems from the early 1980s onwards in an effort to combat soil erosion, whilst at the same time reducing labor, fuel and machinery costs^15^. Moreover, direct seeding without tillage operations allows farmers to plant earlier in the season. This, in combination with the use of shorter-cycle crop varieties (mainly soybean), enabled the planting of a second crop during the same growing season. Today, it is estimated that about 11 million ha of cropland are cultivated under NT systems in the Cerrado (Federação Brasileira de Plantio Direto na Palha, personal communication). The main NT cropping system is soybean followed by a cereal crop, mostly maize. Most studies conducted in the Cerrado reported increased soil C levels with a change from CT to NT^16^,^17^,^18^,^19^,^20^,^21^. Estimated rates of C sequestration in the 0–20 cm soil layer range between −0.03 and 0.83 MgC ha^−1^ yr^−1^. These are average rates for a specific period of measurement that are usually calculated from an increase in soil C levels for a given NT system compared with CT. Several factors may explain the wide range of values, including different soil types, types of cropping systems, and initial soil C stocks. The use of different methodological approaches and experimental set-ups may also have contributed to the variability in estimated soil C sequestration rates.

It has been postulated that rates of soil C sequestration are highest soon after the implementation of NT, and become smaller as the soil C stock approaches a new steady state, i.e. when soil C inputs approximate soil C outputs^22^,^23^. At steady state, the soil C sink is saturated. This also implies that the duration of soil C sequestration is limited i.e. the time period between the old and the new steady state. The Intergovernmental Panel on Climate Change (IPCC) uses twenty years as default value for the duration period of soil C sequestration under improved agricultural management. However, if data on the duration of impact are available, it is recommended to use the appropriate time period, based on country-specific data and measurements^24^. Soils could potentially sequester additional C following new changes in crop management that increase C inputs and/or decrease C outputs. Several studies have, however, suggested that each soil has a maximum soil C storage capacity or C-saturation level that is related to the maximum protective capacity of soil organic carbon (SOC) by soil aggregates and clay minerals^25^,^26^,^27^,^28^. Even though the above limits of soil C sequestration have been illustrated in theoretical modelling studies, they often are overlooked in empirical studies when assessing rates of soil C sequestration under NT systems.

The aim of this study was to quantitatively estimate and analyze (i) the response of soil C stocks to the NT cropping systems in the Cerrado, and (ii) the duration of soil C sequestration under continuous NT systems. The study was conducted on croplands under ‘real-world’ conditions in two municipalities in the Goiás state that are representative of the Cerrado region of Brazil, where farmers have managed continuous NT systems for varying time periods. Studying soil C sequestration ideally requires long-term observations but such studies are difficult to accomplish, especially under farm conditions. A viable, alternative approach is to use the chronosequence or synchronic approach, where plots of different years under NT are sampled simultaneously (space-for-time substitution). This approach provides an integrated assessment of SOC change within a larger area than when e.g. sampling over time one or two experimental fields. In theory, the judicious use of the chronosequence approach should give approximately the same results as the diachronic approach of sampling the same plot repeatedly over time. Chronosequences must however be used with precaution as they are susceptible to the confounding effects of spatial heterogeneity. To control some of these effects, we combined in our study the chronosequence approach with diachronic sampling of the fields for soil C determination. Soil C levels of native Cerrado vegetation and pasture were measured for comparison.

## Results

### Crop carbon inputs

**Figure 1:**
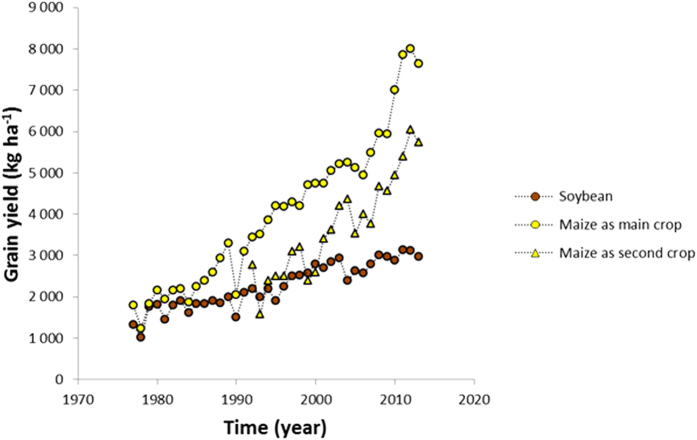
Historical grain yield of soybean and maize (as main or as second crop in the growing season) in the Goiás state, Brazil (data from the Brazilian National Food Supply Agency, www.conab.gov.br).

Historical records (1977–2013) show the positive trends in grain yields of soybean (*Glycine* max L. Merr.) and maize (*Zea mays* L.) in the Goiás state (Fig. 1). These yield increases were the result of various genetic, chemical (pesticide and fertilizer) and mechanical technological innovations. The long-term trends in productivity and, more importantly, changes in cropping intensity (one versus two crops per year) altered above- and belowground crop residue C inputs into the soil. In case of the CT system with typically soybean grown in a 4-year rotation with maize, crop-derived C inputs (harvest residues and roots) to soil were relatively small, because a single crop (soybean or maize) is only present for maximum 4 months of the year. For this ‘historical’ cropping system, we estimated an average C input of 1.5 Mg C ha^−1^ yr^−1^ (between 1977 and 1995). In the new NT systems with two crops per year, i.e. typically soybean followed by maize, estimated crop C inputs were more than tripled (on average 5.3 Mg C ha^−1^ yr^−1^ between 1995 and 2013).

### Soil carbon

#### Soil carbon concentrations over soil depth

**Figure 2:**
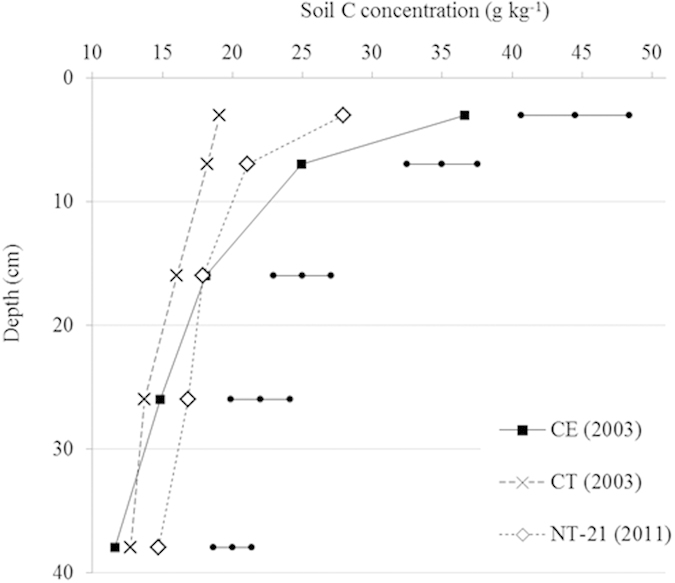
Soil carbon contents as function of soil depth under CE (native Cerrado, 2003 sampling), CT (conventional tillage cropping system, 25 years old, 2003 sampling) and NT-21 (no-tillage cropping system, 21 years old, 2011 sampling). Horizontal lines show the Tukey’s 95% confidence intervals.

Overall, the highest SOC concentrations were measured in the 0–5 cm soil depth layers, and SOC concentrations decreased with increase in soil depth (Fig. 2, Table S3). By far, the strongest stratification by soil depth was observed in CE (native Cerrado vegetation), with SOC concentrations in the 0–5 cm soil layer that were about twice higher than those in the other sites. In long-term NT systems (NT-21), SOC concentrations were significantly (P < 0.05) higher in the 0–5 cm soil layer compared to the 5–10 cm layer, most likely due to accumulation of crop residues on the surface. In contrast, in CT SOC concentrations were comparable in the upper 0–5 and 5–10 cm layers, as a result of incorporation of crop residues into the soil by tillage. Differences in SOC concentrations between NT and CT systems diminished with soil depth, and no significant (P > 0.10) differences were observed in the 30–40 cm layer.

#### Soil carbon sequestration

**Figure 3:**
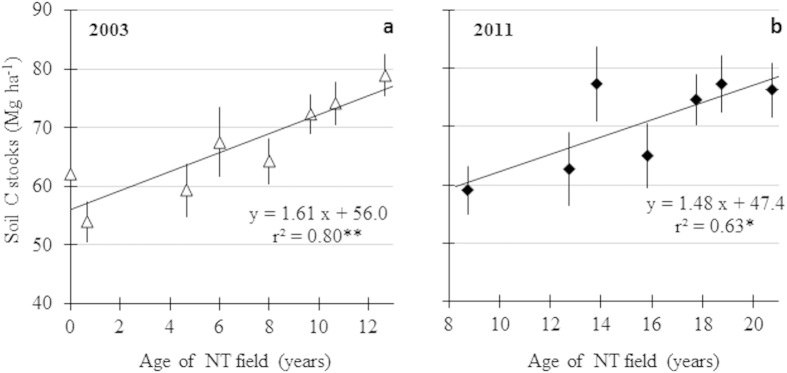
Synchronic assessment (2003 and 2011 sampling) of the mean annual soil C accumulation rates (0–40 cm) in the fields under NT cropping systems –including the CT field as reference in the 2003 sampling. **significant at P < 0.01; *significant at P < 0.05.

In 2003, total SOC stocks on an equivalent mass of soil in the 0–40 cm soil layer were highest in CE (75.3 Mg C ha^−1^) and in the oldest NT fields of 11 and 13 years at that time (NT-11 and NT-13, 74.3 and 78.9 Mg C ha^−1^, respectively), and lowest in the most recent NT fields of 1 and 5 years old (NT-1 and NT-5, 54.0 and 59.4 Mg C ha^−1^, respectively) (Table 1). The fact that the soil C stock in NT-1 was significantly (P < 0.05) lower than that in CT (62.1 Mg C ha^−1^), and this in spite of the shorter CT history of NT-1, was unexpected and suggests that other factors than land management, that are not exactly known to us, interfered. From discussion with the landowner, a possible explanation was the high erosion loss on this field when it was under CT. Differences in soil texture may also be explanatory: the soil under NT-1 contained about 20% less clay + silt than that under CT (Table S2). Soil C stocks increased significantly (P < 0.01) along the chronosequence of 2003 with an average rate of 1.61 Mg C ha^−1^ yr^−1^ (Fig. 3a). In 2011, soil C stocks had reached highest values in CE, PA and the oldest NT fields, NT-14, NT-17, NT-19 and NT-21, with exception of NT-16. The lowest C stock (59.1 Mg C ha^−1^) during the 2011 sampling was observed in the youngest NT field, NT-9. The 2011 chronosequence also showed a significant (P < 0.05) increase in SOC, although with a lower average annual rate than the 2003 chronosequence: 1.48 Mg C ha^−1^ yr^−1^ (Fig. 3b). The soil C stocks under CE between 2003 (75.3 Mg C ha^−1^) and 2011 (76.2 Mg C ha^−1^) were similar, which was according to our expectations, since the natural vegetation is considered to be in steady state, where C inputs to soil equals C outputs. Soil C under pasture increased from 64.3 to 73.2 Mg C ha^−1^, although not significant (P > 0.05). The repeated sampling revealed that younger NT fields tended to show a higher increase in soil C stocks than the older fields, in which little or no change was observed (Table 1). NT-6 (2003 sampling) was the field with the highest increase in soil C between 2003 and 2011, 1.46 Mg C ha^−1^ yr^−1^ (P < 0.01). On the 2 youngest fields (NT-1 (2003) and NT-5 (2003)) the annual soil C accumulation was respectively 0.63 and 0.41 Mg C ha^−1^ yr^−1^ between 2003 and 2011. On older fields annual C accumulation between 2003 and 2011 was lower, ranging from −0.32 to 0.37 Mg C ha^−1^ yr^−1^. Overall, the chronosequence method resulted in higher soil C sequestration rates than the diachronic sampling approach. As indicated above, inevitable differences in soil conditions among the NT fields may, however, have affected results from the chronosequence approach to some extent. On the other hand, SOC stocks change slowly and the cumulative changes after eight years were probably not sufficiently large to be detectable by the diachronic approach.

## Discussion

Conversion of native Cerrado into CT cropland induced a loss in soil C (0–40 cm) of about 17% after 26 years of continuous cultivation of soybean/maize as single crops per season (Table 1, CE and CT, 2003 sampling). A modelling analysis^18^ suggests that these C losses have largely halted and soils under long-term (>25 years) CT are thought to be near steady-state, thus no longer representing a source of atmospheric CO_2_. A number of factors may have contributed to the historical losses in soil C following the clearance of Cerrado for CT cropland, including increased physical soil disturbance, a more favorable soil environment for SOC decomposition, lower C inputs and increased soil erosion. Soil disturbance through tillage decreases soil aggregation resulting in lower protection of SOC from microbial decomposition^27^,^28^. However, probably a more important factor related to the soil disturbance contributing to the soil C decline upon cultivation is increased soil erosion^16^,^29^. In addition, the pH of soils increased typically from 4.7 under native savannah to 5.5 or more under cropland, as a result of periodic lime applications (Table S2). This may also facilitate more rapid SOC decomposition^30^. Finally, the decrease in total C inputs from plants, from an estimated 4.3 Mg C ha^−1^ yr^−1^ under Cerrado savannah^18^ to 1.5 Mg C ha^−1^ yr^−1^ under single soybean or maize cropping, is a major cause for the soil C decline over the long term.

Because the soils under CT have been C depleted over the last 25–30 years, they now represent a potential CO_2_ sink. With adoption of NT systems soil C stocks regained the levels of those under natural Cerrado after about 11–14 years (NT-11 sampled in 2003, NT-14 sampled in 2011, Table 1), representing a total soil C storage of about 15 Mg C ha^−1^ in the 0–40 cm soil layer. SOC levels of NT fields of more than 14 years were not higher than those under natural Cerrado. From this, it is clear that the previous cropping history affects to a large extent the amount of soil C that can be sequestered. Besides, estimated annual rates of soil C sequestration under continuous NT systems tended to decrease with time under NT (Table 1), suggesting that a new steady state was approached, the behavior predicted by classical first-order kinetic soil C models^22^. These models predict that soil C levels accumulate faster and over longer time period if they are far from their steady state level. Our data suggest that after 11 to 14 years of NT management, soil C stocks had reached maximum levels. Thus, it can be inferred that the rate and duration of soil C sequestration with a shift from CT to NT systems depend on the initial soil C in relation to the new steady state, when other management and environmental variables remain constant. In reality, however, other management practices have also changed, e.g. incremental adoption of new crop genotypes, improved fertilization regimes or other agronomic innovations, resulting in a positive trend in crop yields and in rising amounts of C inputs into the soil with time (Fig. 1). This may cause the eventual steady state to shift and the time required to reach the steady state to be longer.

It has also been postulated that there is a maximum amount of C which can be stabilized in soil, and once this capacity is saturated, additional organic material is no longer protected from rapid microbial decomposition and adds little to total soil C storage^4^. The physical capacity of soil to preserve SOC (defined as the maximum amount of C that can be associated with the fine mineral fraction, i.e. <20 Чm clay + fine silt fraction) is limited^25^,^26^. The value of C saturation of the clay size fraction in the Oxisols of the Cerrado has been estimated at 32.5 g C kg^−1^ clay^26^. For Oxisols with a 60% clay content and a soil bulk density of 1.1 Mg m^−3^, this suggests an upper limit in soil C stocks (0–40 cm) of more than 86 Mg C ha^−1^. It indicates that the soils with highest soil C levels in our study have not reached the theoretical upper limit of C saturation.

There is little diversity in the region with regard to the type of crop rotations practiced by the farmers. The dominant cropping system is based on soybean followed by a cereal crop (mainly maize, sometimes sorghum or millet). Crop management in the region is intensive, with high inputs of fertilizers and pesticides. NT management in the Cerrado entailed an increase in soil C inputs, largely through the cultivation of a second crop in the same season. With the cultivation of two crops replacing the single soybean or maize crop we estimated that an extra annual C input of about 3.8 Mg ha^−1^ was produced. Higher crop residue and root inputs may also have synergistic effects on soil C sequestration by enhancing the stabilization of SOC through increased soil aggregation. Besides, double cropping influences the soil temperature and moisture dynamics thereby affecting SOC decomposition, but these effects are believed to be of minor importance. Another aspect is the greater nutrient recycling with double cropping leading to less nutrient leakage, especially nitrogen, which also favors long-term soil C sequestration in agricultural soils^31^. It is also expected that not tilling the soil reduces soil C outputs as a result of lower SOC decomposition. The main effect here is the increased protection of SOC from microbial decomposition through increased soil aggregation with slower turnover rates in untilled soils. This decelerated turnover stimulates the formation and stabilization of more recalcitrant C within micro-aggregates^28^. There are also secondary effects via altered soil temperature and moisture dynamics under NT as a result of changes in soil structure and crop residue mulching. Mulching usually tempers temperature which may lead to lower decomposition rates. On the other hand, it is expected that with mulching more favorable soil moisture conditions for SOC decomposition occur^32^. Part of the observed difference in soil C levels between NT and CT may also have been due to soil erosion^29^. Carbon lost via erosion is, however, not necessarily converted to CO_2_, so that the difference in SOC between NT and CT may overestimate the net soil C sequestration rate under NT systems.Soil organic C increased under the NT systems proportionally more in the upper soil layers compared to the deeper layers (Fig. 2).

In soil under NT systems, SOC is greatest near the surface and declines with increasing soil depth, reflecting surface deposition of crop residues and root distribution, whilst under CT SOC is more evenly distributed within the tilled soil layer. This is a common feature observed for NT systems^5^,^6^,^33^. Consequently, for comparisons of SOC stocks under different tillage systems, soils must be sampled to a depth of at least 30 cm.

Based on our findings, a rough estimation shows that converting an extra 8 million ha of cropland from CT to NT systems in the Cerrado represent a soil C storage of about 8 Tg C yr^−1^ during the first 10 to 15 years. This is equivalent to 29 Tg CO_2_ yr^−1^, which is an important sink given that greenhouse gas emissions in 2010 due to land-use change in the Cerrados were estimated at 113 Tg CO_2_ yr^−1^ 2. It should ,however, be mentioned that the NT effects are reversible, i.e. if management reverts to CT, it is expected that soil C levels will drop again and previously sequestered C will be released as CO_2_^34^.

The full assessment of NT systems as a mitigation option for global warming should also consider additional sources of greenhouse gas emissions, nitrous oxide and methane^35^,^36^, as well as fuel-associated CO_2_ emissions. The few studies that were conducted in the Cerrado indicate overall low N_2_O and CH_4_ emissions from cropland^37^,^38^. Smaller use of agricultural inputs, especially fuel, under NT systems can lead to supplementary reductions in greenhouse gas emissions. On the other hand, greenhouse gas emissions associated with growing the extra crop in the NT systems should also be taken into account, including emissions from the additional N fertilizer used. Overall, it was estimated that fuel use on large scale farms in the Cerrado region can be reduced by up to 50% with NT systems^39^. Changing from CT to NT systems can therefore both enhance soil C sequestration and decrease CO_2_ emissions. While the enhanced soil C sequestration will continue for a limited time, the reduction in net CO_2_ fluxes to the atmosphere caused by the reduced fossil-fuel use can last indefinitely.

Lastly, in many situations NT systems represent a ‘win-win’ situation, where in addition to CO_2_ mitigation other important benefits are achieved, e.g. improved overall soil fertility, protection of land against soil erosion and cost and labor savings^40^. These additional benefits that result often in economic gains for the farmers should be considered as the prime drivers of adoption of NT systems in the Cerrado region, especially as long as no economic instruments are put in place to promote the use of NT as a mitigation strategy of global warming.

## Methods

### Site description

The study area was located in the municipalities of Rio Verde (17° 47′ S, 51° 55′ W) and Montividiu (17° 24′ S, 51° 14′ W) in the south-western part of the Goiás state. The climate of the region is humid tropical of savannah type. Mean annual precipitation is about 1600 mm, with a dry season from May till September. The average monthly temperature varies between 20 °C and 25 °C. The study area covered around 5000 ha of cropland that had been converted from native savannah about 30 years ago, and is representative of the Cerrado plateaus (>750 m a.s.l.) in Central Brazil. The cropland belongs to large-scale commercial farmers with farm sizes of between 500 and 2000 ha. Past (before 1990) cropping consisted of a single crop (soybean or maize) with the use of a harrow disk as the main tillage implement (in this study this is referred to as CT). Tillage depth was about 20 cm with the major part of the crop residues buried into the soil. NT was introduced in the region in 1990 and since then an area-wide adoption occurred. NT allowed the cultivation of a second commercial crop (maize or sorghum) or a cover crop (pearl millet) following the main crop (mainly soybean). The native vegetation in the study area is classified as Cerrado *sensu stricto* (tree dominated scrub of shrubs and trees of 3–8 m height with grass understory) and Cerradão (dry semi deciduous woodland)^41^.

In 2002, seven fields belonging to different farms were identified^42^ to represent a chronosequence of sites that were at that time respectively 0 (meaning that the practice of NT cropping started in 2002), 4, 5, 7, 8, 10 and 12 years under continuous NT. In addition, a field under conventional tillage (CT), a permanent pasture of 17 years old (PA, *Brachiaria decumbens (Stapf)*.) and a plot under native Cerrado vegetation (CE) were included as references of, respectively, the traditional tillage-based cropping system, the extensive cattle grazing system, and the original natural ecosystem. In 2002, the CT field had been under conventional tillage for 25 years. All NT fields had a comparable cropping history before they were converted from CT to NT (Table S1). They were established on former native savannah vegetation between 1977 and 1991, and had been cropped under soybean and/or maize (one crop per year) using CT during at least 7 years prior to NT adoption. All sites (including the PA and CE plots) had the same soil type –red yellow Latosol (Brazilian soil classification) or Typic Acrustox (USDA soil taxonomy) – with clay plus silt content ranging from 550 to 730 g kg^−1^. The soil color^43^ corresponded in all sites to the 2.5YR matrix that is characteristic for the red yellow Latosols of the Cerrado. Selected physico-chemical soil properties are given in Table S2. The mineral composition of the soils showed also a clear similarity in all sites, the main minerals being quartz (SiO_4_), kaolinite (Al_2_Si_2_O_5_(OH)_4_), gypsite (Al(OH)_3_), hematite (Fe_2_O_3_) and titanium oxide (TiO_2_) (data not shown). All fields were situated on the plateau of the landscape with slopes of 3 to 5%.

### Soil sampling and analyses

In 2003, a first soil sampling of the sites was carried out in three geo-referenced sub-areas (10 × 20 m) of each field of the above chronosequence and in the CT, PA and CE plots. The NT fields were named as follows: NT-1, NT-5, NT-6, NT-8, NT-9, NT-11 and NT-13, respectively. The NT-1 field corresponds to a practice of NT cropping that started in 2002 while on the NT-13 field NT cropping commenced in 1990. In each sub-area undisturbed samples from the 0–5, 5–10, 10–20, 20–30, 30–40 cm soil layers were collected at six different points and analyzed separately. All soil samples were collected with stainless steel cylinders. Two cylinders of 5 cm height and 8.5 cm diameter (253.7 cm^3^) were used for the upper layers, 0–5 and 5–10 cm, and one cylinder of 10 cm height and 8.5 cm diameter (567.5 cm^3^) for the layers 10–20, 20–30 and 30–40 cm, resulting in similar volumes of soil for each layer sampled. Soil bulk density was determined on field-moist soil. The soil samples were then oven-dried for several days, homogenized and manually crushed to pass through a 2 mm sieve. All visible plant material larger than the 2 mm sieve size was removed. Soil particle-size analysis was determined by densitometry after aggregate dispersion with hexametaphosphate and digestion of the organic material in H_2_O_2_^44^. Sub-samples from the 2-mm sieved soil were finely ground (<150 mm) using a stainless steel ball-mill grinder before analysis for organic C by dry combustion in a LECO CN-2000 analyzer.

In 2011, we re-sampled all fields of the chronosequence as well as the PA and CE plots. As this second sampling was done 8 years after the pervious sampling in 2003, we renamed the NT fields as follows NT-9, NT-13, NT-14, NT-16, NT-17, NT-19 and NT-21, thereby referring to the age of the NT fields in 2011. The CT field was in 2011 no longer cultivated under CT. The exact cropping history since the 2003 sampling of this field could not be established with the landowner, since he had rented out his land to other farmers. Soil sampling in 2011 was carried out in the same sub-areas of the sites and following the same methods as in 2003.

### Calculations

Soil organic C stocks were calculated from the organic C concentrations and soil bulk densities in each sampled layer. To correct for differences in soil bulk density, SOC stocks were estimated based on an equivalent soil mass-depth basis^45^,^46^ that is, as the total C content of the same weight of soil as that present to 40-cm depth of the situation under native Cerrado vegetation (CE, the reference area). Rates of soil C sequestration were calculated following two methods. In the first method, rates were calculated from changes of soil C stocks over real time, i.e. from the soil samples that were taken in 2003 and 2011 on the same location of the different sites (diachronic approach). In the second approach, rates were calculated from C stocks determined on soil samples that were taken from the fields of the chronosequence (synchronic approach). With this approach, where space is substituted for time, it is assumed that the initial soil conditions of the fields under the different land-use or management systems are similar and, in fact, that all influences other than land-use or management are excluded. The fields in our study were carefully selected in order to minimize non-wanted sources of soil C variation, including differences in soil texture, soil mineralogy and the slope of the field.

Average annual above- and belowground crop residue C inputs to soil for the CT and NT cropping systems were estimated on the basis of historical records of grain yields and by using crop-specific harvest indices and root:shoot ratios from published biomass partitioning studies. Average farmers’ yields of soybean and maize (as the main and second crop) in the Goiás state for the period 1977–2013 were retrieved from the database of the Brazilian National Food Supply Agency (CONAB) (www.conab.gov.br). We assumed an average and constant harvest index of 0.45 for maize^47^ and 0.50 for soybean^48^. A conservative value for the root:shoot ratios -corresponding to the 0–40 cm layer- of maize and soybean was assumed at 0.35^49^. Carbon inputs were then calculated by multiplying the residue biomass values by 43%.

### Statistical analyses

A linear mixed model was used to test the effect of site (fixed effect) on soil C stocks (0–40 cm), independently for the two sampling years (2003 and 2011). The replicated measurements in the three sub-areas of each field were entered as a random factor. A covariance analysis using the general linear model procedure revealed no significant effects of clay content and clay + silt content as covariates and no site–covariate interactions. For testing the effect of site and depth on soil bulk densities and soil C concentrations for each sampling year, an analysis of variance was performed using a nested linear mixed model with soil depth nested within site. The Tukey’s test was used to test for significant pairwise differences at a 5% significance level. To compare the means of soil bulk density, soil C concentration (per site and soil depth) and soil C stock (per site) between the two sampling years (2003 and 2011) the Student’s t-test for paired samples was applied. Simple linear regressions of field-average soil C stock versus age of NT fields were performed to assess temporal changes in soil C with the chronosequence approach. All statistical analyses were run using the Statistical Analysis System (SAS), version 9.1.2.

## Additional Information

**How to cite this article**: Corbeels, M. *et al*. Evidence of limited carbon sequestration in soils under no-tillage systems in the Cerrado of Brazil. *Sci. Rep*. **6**, 21450; doi: 10.1038/srep21450 (2016).

## Supplementary Material

Supplementary Information

## Figures and Tables

**Table 1:** Measured soil carbon stocks (0–40 cm) and calculated diachronic annual soil C accumulation rates at the sampled sites.

Site	Soil C stock^1^ (Mg C ha^−1^)			Diachronic annual C accumulation^2^ (Mg C ha^−1^ year^−1^)
	2003	Site	2011	ΔC
CE	75.3 ± 1.6^a^	CE	76.2 ± 1.7^a^	0.11^ns^ (−0.6–0.6)
PA	64.3 ± 0.9^c^	PA	73.2 ± 1.3^ab^	1.10^ns^ (0.3–1.8)
CT	62.1 ± 0.8^c^	CT	–	–
NT-1	54.0 ± 0.8^d^	NT-9	59.1 ± 1.0^d^	0.63^ns^ (−0.1–0.8)
NT-5	59.4 ± 1.1^cd^	NT-13	62.7 ± 1.5^c^	0.41^ns^ (−0.2–0.8)
NT-6	67.5 ± 1.4^bc^	NT-14	77.4 ± 0.8^a^	1.46** (0.6–1.8)
NT-8	65.1 ± 1.2^c^	NT-16	65.8 ± 2.1^c^	0.09^ns^ (−0.4–0.6)
NT-9	72.3 ± 0.8^b^	NT-17	74.6 ± 1.0^ab^	0.28^ns^ (−0.1–0.7)
NT-11	74.3 ± 0.9^ab^	NT-19	77.3 ± 1.1^a^	0.37^ns^ (−0.1–0.8)
NT-13	78.9 ± 0.8^a^	NT-21	76.3 ± 1.1^a^	−0.32^ns^ (−0.6–0.1)

CE, native Cerrado; PA, pasture; CT, conventional tillage cropping system; NT, no-tillage cropping system, with the numbers referring to the age of the NT fields in 2003 and 2011.

^1^Mean (n = 18) ± SD;

^2^Mean (range); Within sampling year, means followed by the same letter are not significantly different at P < 0.05 (Tukey’s test). Between sampling years, **significantly different at P < 0.01, ns = not significantly different at P > 0.05 (Student’s t-test).
