# Supplementary material for: Evidence of limited carbon sequestration in soils under no-tillage systems in the Cerrado of Brazil
Source: Sci Rep. 2016 Feb 24;6:21450. doi: 10.1038/srep21450 (PMC4764901; doi:10.1038/srep21450)
Supplement: Supplementary Information [file srep21450-s1.doc]

**SuPPLEMENTARY INFORMATION**

Correspondence and requests for materials

**Evidence of limited carbon sequestration in soils under no-tillage systems in the Cerrado of Brazil**

Marc Corbeels^a,b,*^, Robelio Leandro Marchão^b^, Marcos Siqueira Neto^c^, Eliann Garcia Ferreira^d^, Beata Emöke Madari^e^, Eric Scopel^a^ and Osmar Rodrigues Brito^d^

^a^ Agro-ecology and Sustainable Intensification of Annual Crops, CIRAD, Avenue Agropolis, 34398 Montpellier cedex 5, France

^b^ Embrapa-Cerrados, PO Box 8233, 73301-970 Planaltina, DF, Brazil

^c^ Centro de Energia Nuclear na Agricultura, Universidade de São Paulo, PO Box 96, 13400-970, Piracicaba, SP, Brazil

^d^ Universidade Estadual de Londrina, Londrina, Brazil

^e^ Embrapa Arroz e Feijão PO Box 179, 75375-000, Santo Antônio de Goiás, GO, Brazil

*Corresponding author:

Marc Corbeels

e-mail: corbeels@cirad.fr

phone: +55 61 91974061

Table S1. Location and historical land use/management practices of the sampled sites.

| Site | Latitude | Longitude | Historical land use/management |
| --- | --- | --- | --- |
| CE | 17°39’07”S | 51°02’49”W | Cerrado native vegetation |
| PA | 17°38’58”S | 51°03’57”W | 1978 – deforestation; 1978 – CT with Ri; 1985 – pasture (reformed in 2007) |
| CT | 17°41’36”S | 51°10’56”W | 1977 – deforestation; 1977 – CT with Ri; 1979 – CT with Sb/Mz; (from 2008 onwards – NT with Sb/So) |
| NT-1 (NT-9) | 17°36’50”S | 51°08’31”W | 1990 – deforestation; 1990 – CT with Ri; 1992 – CT with Sb/Mz ; 2002 – NT with Sb and Mz/So/Mi |
| NT-5 (NT-13) | 17°36’57”S | 51°08’27”W | 1990 – deforestation; 1990 – CT with Ri; 1992 – CT with Sb/Mz; 1998 – NT with Sb and Mz/So/Mi |
| NT-6 (NT-14) | 17°27’54”S | 51°19’55”W | 1991 – deforestation; 1991 – CT with Ri; 1992 – CT with Sb; 1998 – NT with Sb/Mz |
| NT-8 (NT-16) | 17°39’00”S | 51°03’28”W | 1983 – deforestation; 1983 – CT with Ri; 1986 – CT with Sb/Mz; 1995 – NT with Sb and Mz/Mi |
| NT-9 (NT-17) | 17°30’11”S | 51°09’57”W | 1981 – deforestation; 1981 – CT with Ri; 1985 – CT with Sb/Mz; 1994 – NT with Sb and Mz/So/Mi |
| NT-11 (NT-19) | 17°28’16”S | 51°16’19”W | 1977 – deforestation; 1978 – pasture; 1983 – CT with Ri; 1985 – CT with Sb/Mz; 1992 – NT with Sb/Mz and Mz/So/Mi |
| NT-13 (NT-21) | 17°20’12”S | 51°15’03”W | 1977 – deforestation; 1978 – pasture; 1982 – CT with Ri; 1984 – CT with Sb/Mz ; 1990 – NT with Sb/Mz and Mz/So/Mi |

CE, native Cerrado; PA, pasture; CT, conventional tillage; NT, no-tillage, with the numbers referring to the age of the NT-fields in 2003 and 2011 (in brackets); Mi = millet; Mz = maize; Ri = rice; Sb = soybean; So = sorghum.

The management history of the fields was retrieved from current landholders.

Table S2. Selected physic-chemical characteristics of the soils (0-20 cm) of the sampled sites under the different land use/management practices (sampled in 2003).

| Site | pH (H_2_0) | | clay | | silt | sand | | Available-P  - Mehlich - | | | CEC | | | BS | | |  |
| --- | --- | --- | --- | --- | --- | --- | --- | --- | --- | --- | --- | --- | --- | --- | --- | --- | --- |
|  |  |  | | % | | |  | | mg kg^-1^ |  | | cmolc dm^-3^ |  | | % |  | |
| CE | 4.7 ± 0.2 | | 50.2 ± 2.6 | | 3.0 ± 1.4 | 46.8 ± 2.8 | | 6.8 ± 3.7 | | | 8.3 ± 0.8 | | 5.0± 3.3 | | | |  |
| PA | 6.0 ± 0.1 | | 65.3 ± 3.0 | | 4.6 ± 1.7 | 30.1 ± 3.2 | | 8.3 ± 3.5 | | | 5.3 ± 0.4 | | 43.0 ± 17.8 | | | |  |
| CT | 5.6 ± 0.2 | | 61.7 ± 3.4 | | 5.8 ± 1.4 | 32.4 ± 3.6 | | 11.3 ± 7.6 | | | 5.1 ± 0.4 | | 27.6 ± 12.6 | | | |  |
| NT-1 | 5.9 ± 0.3 | | 52.0 ± 2.2 | | 3.2 ± 1.2 | 44.8 ± 2.1 | | 40.8 ± 9.9 | | | 5.8 ± 0.4 | | 44.2 ± 12.0 | | | |  |
| NT-5 | 5.8 ± 0.3 | | 58.9 ± 2.7 | | 3.2 ± 1.2 | 37.9 ± 3.1 | | 27.2 ± 13.3 | | | 5.8 ± 0.2 | | 40.1 ± 10.3 | | | |  |
| NT-6 | 5.8 ± 0.2 | | 60.8 ± 2.1 | | 4.0 ± 1.3 | 35.2 ± 2.6 | | 25.2 ± 6.3 | | | 5.6 ± 0.5 | | 32.0 ± 17.1 | | | |  |
| NT-8 | 5.9 ± 0.3 | | 65.4 ± 3.5 | | 5.2 ± 1.7 | 29.4 ± 2.4 | | 11.6 ± 6.5 | | | 5.9 ± 0.2 | | 41.9 ± 11.5 | | | |  |
| NT-9 | 5.6 ± 0.3 | | 68.1 ± 4.3 | | 5.2 ± 2.3 | 26.7 ± 3.7 | | 16.0 ± 5.7 | | | 6.0 ± 0.7 | | 34.5 ± 13.9 | | | |  |
| NT-11 | 5.5 ± 0.2 | | 65.0 ± 5.3 | | 6.9 ± 3.1 | 28.1 ± 3.5 | | 16.1 ± 5.8 | | | 6.6 ± 0.8 | | 30.0 ± 9.7 | | | |  |
| NT-13 | 5.9 ± 0.4 | | 64.3 ± 4.2 | | 5.2 ± 2.1 | 30.6 ± 4.0 | | 29.3 ± 9.3 | | | 5.6 ± 1.1 | | 27.9 ± 11.6 | | | |  |

Mean (n = 6) ± S.D.; CE, native Cerrado; CT, conventional tillage; NT, no-tillage, with the numbers referring to the age of the NT-fields in 2003; P, phosphorus; CEC, cation exchange capacity; BS, base saturation.

Soil pH was measured in a 1:2.5 (soil:water) extract. Clay and silt contents were determined by densitometry after aggregate dispersion with hexametaphosphate and digestion of the organic material in H_2_O_2_. Available P and exchangeable cations (K^+^, Ca^2+^ and Mg^2+^) were extracted with ion exchange resins (Van Raij & Quaggio, 1983). P was determined by visible spectrophotometry and K^+^ by flame spectrophotometry. Ca^2+^ and Mg^2+^ were determined by atomic absorption spectrophotometry. Potential acidity (H^+^ + Al^3+^) was determined using 1N calcium acetate solution at pH 7.0 (Embrapa, 1997). Cation exchange capacity (CEC) at pH 7.0 and base saturation (BS) were obtained by the sum of the exchangeable cations (Ca^2+^, Mg^2+^, K^+^) and (H^+^+ Al^3+^).

Embrapa Manual de Métodos de Análise do Solo. Empresa Brasileira de Pesquisa Agropecuária, Centro Nacional de Pesquisa de Solos, Rio de Janeiro. (1979)

Van Raij, B. & Quaggio, J.A. Métodos de análise de solo para fins de fertilidade. Instituto Agronômico de Campinas. 16p (1983).

Table S3. Soil bulk density and C concentration of soil layers of the sampled sites under the different land use/management practices (sampled in 2003 and 2011).

| Site | Soil bulk density (g cm^-3^) | | | | | | | | | | | | | | | | | | | |
| --- | --- | --- | --- | --- | --- | --- | --- | --- | --- | --- | --- | --- | --- | --- | --- | --- | --- | --- | --- | --- |
|  |  | 0–5 cm | |  | | 5–10 cm | |  | | 10–20 cm | |  | | 20–30 cm | |  | | 30–40 cm | |  |
|  | 2003 | | 2011 | | 2003 | | 2011 | | 2003 | | 2011 | | 2003 | | 2011 | | 2003 | | 2011 | |
| CE | 0.87^d - ns^ | | 0.84^d^ | | 1.01^c^ ^ns^ | | 1.00^c^ | | 1.19^b - **^ | | 1.06^d^ | | 1.20^ab - **^ | | 1.06^d^ | | 1.23^a - **^ | | 1.06^c^ | |
| PA | 1.23^a- ns^ | | 1.25^a^ | | 1.25^a - ns^ | | 1.20^ab^ | | 1.19^bc - ns^ | | 1.18^b^ | | 1.23^a - ns^ | | 1.19^bc^ | | 1.21^ab - ns^ | | 1.21^b^ | |
| CT | 1.03^bc - **^ | | - | | 1.11^b - **^ | | - | | 1.14^bc - **^ | | - | | 1.13^bc - **^ | | - | | 1.11^bc - *^ | | - | |
| NT-1 | 1.15^ab - *^ | | 1.27^a^ | | 1.20^ab - **^ | | 1.26^a^ | | 1.22^ab - *^ | | 1.30^a^ | | 1.26^a - ns^ | | 1.29^a^ | | 1.24^a - ns^ | | 1.26^a^ | |
| NT-5 | 1.13^ab - ns^ | | 1.18^ab^ | | 1.17^ab - ns^ | | 1.22^ab^ | | 1.18^bc - ns^ | | 1.22^b^ | | 1.19^b - **^ | | 1.24^b^ | | 1.17^b - ns^ | | 1.24^ab^ | |
| NT-6 | 1.07^b - *^ | | 1.04^cd^ | | 1.14^bc - ns^ | | 1.15^b^ | | 1.22^ab - **^ | | 1.17^b^ | | 1.18^b - ns^ | | 1.17^c^ | | 1.22^ab - *^ | | 1.18^b^ | |
| NT-8 | 1.11^ab - *^ | | 1.17^ab^ | | 1.20^a - **^ | | 1.27^a^ | | 1.28^a - ns^ | | 1.23^b^ | | 1.22^a - ns^ | | 1.22^bc^ | | 1.26^a - **^ | | 1.18^b^ | |
| NT-9 | 0.92^cd - **^ | | 1.05^bc^ | | 1.04^c - **^ | | 1.17^b^ | | 1.09^c - **^ | | 1.16^bc^ | | 1.07^c - **^ | | 1.15^c^ | | 1.07^c - **^ | | 1.18^b^ | |
| NT-11 | 0.97^cd - **^ | | 1.14^b^ | | 1.04^c - **^ | | 1.21^ab^ | | 1.11^c - **^ | | 1.23^b^ | | 1.07^c - **^ | | 1.24^b^ | | 1.05^c - **^ | | 1.23^ab^ | |
| NT-13 | 0.96^cd - *^ | | 1.05^bc^ | | 1.04^c - **^ | | 1.13^bc^ | | 1.11^c - ns^ | | 1.15^bc^ | | 1.10^bc - **^ | | 1.18^bc^ | | 1.09^c - **^ | | 1.18^b^ | |
|  | Soil C concentration (g kg^-1^) | | | | | | | | | | | | | | | | | | | |
| CE | 36.6^a - ns^ | | 37.8^a^ | | 24.9^a - ns^ | | 23.5^a^ | | 18.1^a - **^ | | 19.2^a^ | | 14.8^b - ns^ | | 16.4^a^ | | 11.6^c - **^ | | 13.9^b^ | |
| PA | 20.7^b - ns^ | | 24.2^b^ | | 19.0^bc - ns^ | | 20.5^bc^ | | 16.4^b - *^ | | 17.9^b^ | | 13.2^c - ns^ | | 15.9^b^ | | 11.5^c - *^ | | 14.0^ab^ | |
| CT | 19.1^cd - **^ | | - | | 18.2^bc - **^ | | - | | 16.0^bc - ns^ | | - | | 13.7^c - **^ | | - | | 12.7^b - *^ | | - | |
| NT-1 | 17.0^d - **^ | | 19.2^d^ | | 15.3^c - ns^ | | 15.4^d^ | | 13.3^cd - ns^ | | 14.2^c^ | | 12.0^c - ns^ | | 13.2^c^ | | 10.7^c - **^ | | 12.0^b^ | |
| NT-5 | 19.4^cd - **^ | | 21.2^cd^ | | 16.7^c - ns^ | | 16.6^d^ | | 14.9^c - *^ | | 15.1^bc^ | | 13.1^b - ns^ | | 13.9^c^ | | 11.5^c - **^ | | 13.5^b^ | |
| NT-6 | 21.4^bc - *^ | | 26.9^ab^ | | 18.6^bc - **^ | | 21.0^bc^ | | 17.6^b - ns^ | | 19.2^a^ | | 15.2^b - *^ | | 17.5^a^ | | 12.6^bc - **^ | | 12.2^b^ | |
| NT-8 | 22.8^bc - ns^ | | 23.2^bc^ | | 17.7^c - ns^ | | 17.2^cd^ | | 17.1^b - ns^ | | 16.0b | | 13.4^c - ns^ | | 14.4^bc^ | | 11.1^c - **^ | | 14.2^ab^ | |
| NT-9 | 24.2^b - ns^ | | 26.6^b^ | | 20.6^b - ns^ | | 20.6^bc^ | | 19.8^a - ns^ | | 18.7^a^ | | 16.4^a - ns^ | | 16.4^a^ | | 13.4^b - *^ | | 15.2^a^ | |
| NT-11 | 24.7^b - ns^ | | 26.7^ab^ | | 20.7^b - **^ | | 21.3^b^ | | 19.0^a - ns^ | | 18.9^a^ | | 17.0^a - ns^ | | 16.6^a^ | | 15.0^a - *^ | | 14.7^a^ | |
| NT-13 | 27.8^ab - ns^ | | 27.9^ab^ | | 22.5^ab - ns^ | | 21.1^ab^ | | 20.8^a - *^ | | 17.9^b^ | | 17.0^a - ns^ | | 16.8^a^ | | 15.4^a - ns^ | | 14.7^a^ | |

^1^Mean (n = 18); within sampling year, means followed by the same letter are not significantly different at P < 0.05 (Tukey’s test). Between sampling years, * = significantly different at P < 0.05, ** at P < 0.01, ns = not significantly different (t-test).

CE, native Cerrado; PA, pasture; CT, conventional tillage; NT, no-tillage, with the numbers referring to the age of the NT-fields in 2003
